# Multi-Scale Investigation of Carbonation Evolution and Microstructural Changes in Concrete Containing Fly Ash and Silica Fume

**DOI:** 10.3390/ma19112426

**Published:** 2026-06-05

**Authors:** Jianghuai Zhan, Lepeng Huang, Tiansheng Shang, Xuanyi Xue, Jing Li, Shuai Li, Jianmin Hua, Jilin Song

**Affiliations:** 1 School of Civil Engineering, Chongqing University, Chongqing 400045, China; 2 State Key Laboratory of Safety and Resilience of Civil Engineering in Mountain Area, Chongqing 400045, China; 3 Support and Service Center of China Academy of Engineering Physics, Mianyang 621999, China; 4 Laser Fusion Research Center, China Academy of Engineering Physics, Mianyang 621999, China; 5 Department of Civil Engineering, The University of Hong Kong, Pokfulam Road, Hong Kong 999077, China; 6 China Construction Third Engineering Bureau Group Co., Ltd., Wuhan 430064, China

**Keywords:** low-carbon concrete, mechanical properties, carbonation, nanoindentation, microstructure

## Abstract

This study systematically investigated the durability of low-carbon concrete under severe service conditions using industrial solid wastes. The mechanical properties and carbonation resistance (including carbonation depth, compressive strength after carbonation, and splitting tensile strength after carbonation) were tested. Multi-scale characterization techniques, including XRD, SEM-EDS, and nanoindentation, were employed to investigate the microstructure. This approach revealed a synergistic mechanism linking microstructural evolution to the concrete’s macroscopic mechanical and durability performance. Results showed that incorporating 25% fly ash (FA) reduced compressive strength by 11.30% and 11.39% in CF-25 and BF-25 mixes, respectively, and increased carbonation depth by 58.46% in CF-25. In contrast, the addition of 5% silica fume (SF) produced different effects. It significantly enhanced the compressive strength of the CS-5 and BS-5 mixes by 18.92% and 9.94%, respectively. Furthermore, it improved the micromechanical properties of the interfacial transition zone (ITZ) and reduced its thickness. Micro-mechanistic analysis revealed that the low pozzolanic activity of FA at early ages led to insufficient hydration products, higher porosity, and a weaker ITZ. Conversely, SF, through its high pozzolanic reactivity and nano-filling effect, promoted a dense, highly polymerized gel structure and optimized pore size distribution. The distinct chemical characteristics of high-calcium and low-calcium cementitious systems further amplified the differential effects of these supplementary materials.

## 1. Introduction

As the most widely used artificial construction material globally, concrete relies on ordinary Portland cement as its core component. However, the production of this traditional cement is accompanied by high energy consumption and significant carbon emissions, posing a major constraint to achieving the strategic “dual carbon” goals [1,2]. At present, the decarbonization pathway primarily relying on incorporating supplementary cementitious materials into conventional cement has encountered bottlenecks, necessitating a fundamental transformation of the binder system. Novel silica-alumina-based low-carbon cement that utilize industrial solid wastes (including fly ash, slag, and steel slag) as main raw materials offer a promising alternative [3]. Through mechanical activation and chemical stimulation techniques, these materials can substantially reduce or even eliminate limestone calcination, thereby lowering carbon emissions at the source and demonstrating notable environmental advantages [4,5]. Nevertheless, the synergistic effects between such cements and traditional supplementary materials like FA and silica fume (SF), as well as the long-term performance and dynamic mechanical responses of the resulting concrete, remain insufficiently studied. This knowledge gap seriously hinders the standardization and large-scale engineering application of these innovative materials.

Achieving low-carbon concrete fundamentally requires innovation in the cementitious system. The conventional approach primarily relies on utilizing industrial by-products such as FA and SF as supplementary cementitious materials to partially replace cement [6]. FA, a solid waste from coal-fired power plants, exhibits a pozzolanic effect, reacting with calcium hydroxide—a product of cement hydration—to form additional cementitious C-S-H gel. This process enhances the long-term strength, reduces the heat of hydration, and improves the durability of concrete [7]. However, the low early-age reactivity of FA often results in slower strength development at early stages. SF, an ultra-fine powder recovered from the production of ferrosilicon and metallic silicon, possesses a high silica content and a large specific surface area. These characteristics confer a significant pozzolanic effect and a micro-aggregate filling effect, enabling it to effectively refine the pore structure of concrete [8] and substantially enhance both strength and durability [9,10]. Lv et al. [11] found that SF exhibited a significantly superior effect compared to FA in enhancing the abrasion resistance of materials. In the GS5 material containing 5% SF, the abrasion strength and impact energy were 18.5% and 37.9% higher, respectively, than those of the GF25 material containing 25% FA. Wang et al. [12] observed that the influence of SF incorporation on the compressive strength of geopolymer-based ultra-high performance concrete (UHPC) was complex and strongly dependent on the calcium aluminate cement content. In systems with a calcium aluminate cement content of 20%, the compressive strength increased with higher SF dosages; however, in systems with a calcium aluminate cement content of 10%, higher SF dosages (>10%) adversely affected the compressive strength. Chen et al. [13] reported that the addition of FA and SF refined the pore structure, reduced permeability, and delayed degradation, resulting in a slower deterioration rate, slower porosity increase, and a longer service life for the blended concrete compared to ordinary cement concrete. Mudasir Nazeer et al. [6] demonstrated that replacing 10% of ordinary Portland cement with a combination of FA and SF led to significant improvements in both the strength and durability of concrete. At 28, 56, and 120 d, the compressive strength showed notable increases of 41%, 85%, and 94%, respectively. Nejib Ghazouani et al. [14] found that with 30% SF replacement, the prepared ultra-high performance green cementitious composite achieved a 28-day compressive strength of 134.5 MPa. The SF mix also exhibited the lowest capillary water absorption coefficient (0.16758 mm/min½). Drying shrinkage decreased with increasing SF content, reaching a minimum value of 4000 μm/m. Thermal analysis indicated lower calcium hydroxide content and more intensive secondary hydration reactions in the SF mixes. Qiu et al. [15] highlighted that the fine particle size and high reactivity of FA and SF facilitated pore filling and generated additional C-S-H gel during the hydration process. This not only improved the pore structure of the mortar but also strengthened the interfacial transition zone around the coal gangue aggregates.

The durability and safety of concrete structures are critical factors determining their service life, particularly in harsh coastal environments. Carbonation resistance serves as a key indicator for evaluating concrete durability: atmospheric carbon dioxide permeates the concrete interior and neutralizes alkaline hydration products, lowering the pH of the pore solution. This process directly damages the passive film on reinforcing steel surfaces, creating conditions for chloride ion-induced corrosion [16]. Therefore, systematically assessing the carbonation resistance of low-carbon cement is essential for predicting its ability to protect reinforcement and estimating its service life. Given that the low-carbon cement system may differ from traditional cement in terms of hydration product composition, pore structure characteristics, and moisture transport mechanisms, an in-depth investigation of its carbonation behavior is crucial for evaluating the durability of concrete produced with this system [17].

To gain a deeper understanding of the micro-mechanisms underlying the differences in macroscopic performance of low-carbon concrete, and to address the research gap that existing literature has primarily focused on traditional Portland cement systems or individual admixture effects while lacking systematic investigation of carbonation behavior in silica-alumina-based low-carbon cement incorporating both FA and SF, this study systematically investigated the effects of incorporating 25% fly ash and 5% silica fume into ordinary Portland cement and low-carbon cement matrices. A total of six mix proportions were designed with a constant water-to-binder ratio of 0.3. The mechanical properties and carbonation depth of each group were systematically tested. Multi-scale characterization techniques, including nanoindentation, X-ray diffraction (XRD), and scanning electron microscopy coupled with energy-dispersive spectroscopy (SEM-EDS), were comprehensively employed to elucidate the micro-mechanisms underlying macroscopic performance evolution. These elucidations were made from the perspectives of interfacial transition zone (ITZ) characteristics, hydration product composition, and pore structure evolution. This study advances the understanding of carbonation behavior in low-carbon concrete in the following three aspects: (1) In contrast to ordinary Portland cement systems, this study focuses on a low-calcium silica-alumina-based binder system, revealing how the absence of abundant portlandite alters the carbonation mechanism; (2) By comparing the effects of 25% fly ash and 5% silica fume, the distinct roles of the two admixtures are clarified—silica fume enhances carbonation resistance through pore refinement and additional C-S-H gel formation, whereas excessive fly ash accelerates carbonation due to a reduced Ca/Si ratio and increased porosity; (3) Through multi-scale macro-micro characterization, this study establishes, for the first time, the intrinsic correlation between carbonation depth and microstructural parameters such as interfacial transition zone characteristics, hydration product composition, and pore structure evolution in silica-alumina-based low-carbon concrete. The principal innovations of this study are as follows: a novel composite cementitious system was developed using silica–alumina-based low-carbon cement as the primary binder, thereby transcending the traditional carbon reduction paradigm of “Portland cement + mineral admixtures”; the carbonation mechanism of low-carbon cement concrete, along with its intrinsic correlation with microstructural features, was systematically elucidated through multi-scale macro-micro characterization; and an investigation of carbonation resistance as a critical durability indicator was undertaken, filling the research gap in the study of the long-term service performance of low-carbon cement concrete. These findings not only answer how the current study advances the understanding of carbonation behavior in silica-alumina-based low-carbon cement but also provide actionable guidance for optimizing FA and SF dosages to balance mechanical performance and carbonation resistance. The findings are expected to provide critical theoretical and data-driven support for the performance regulation and engineering application of low-carbon concrete, facilitating its transition from laboratory research to engineering practice and contributing to the green and sustainable development of the construction industry.

## 2. Materials and Experimental Methods

### 2.1. Materials

**Figure 1:**
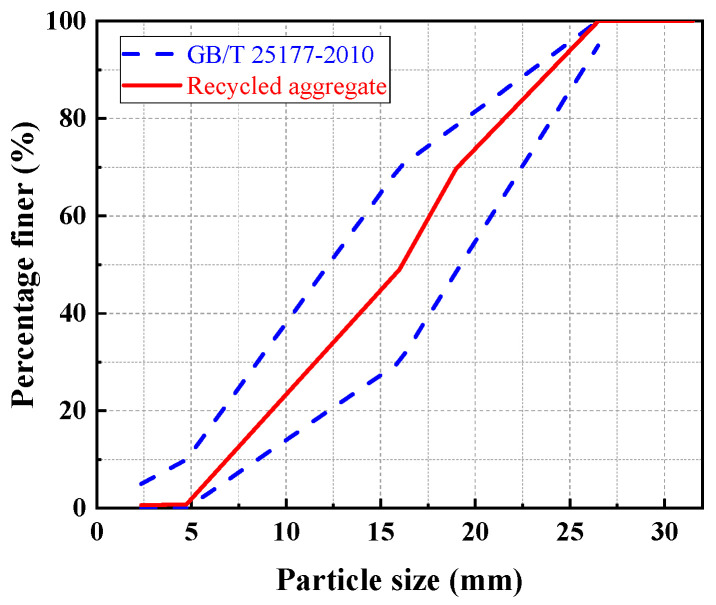
Particle size distribution curve of recycled coarse aggregate [18].

**Figure 2:**
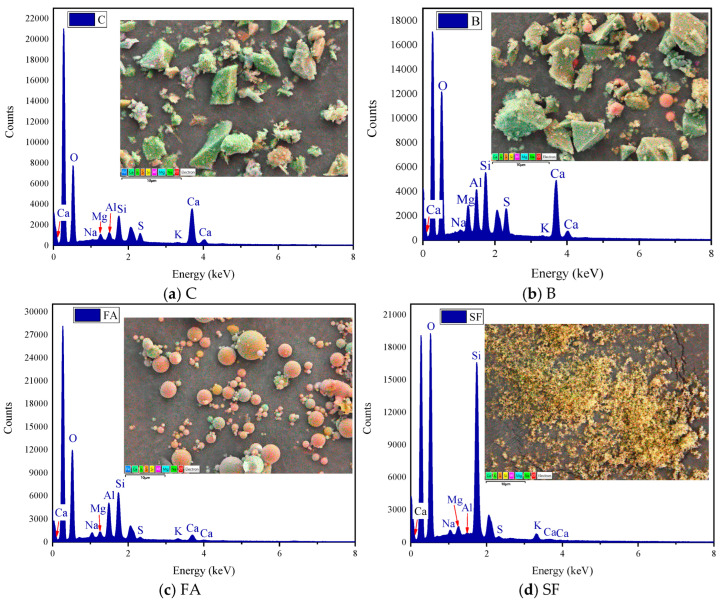
SEM-EDS images of raw materials.

**Figure 3:**
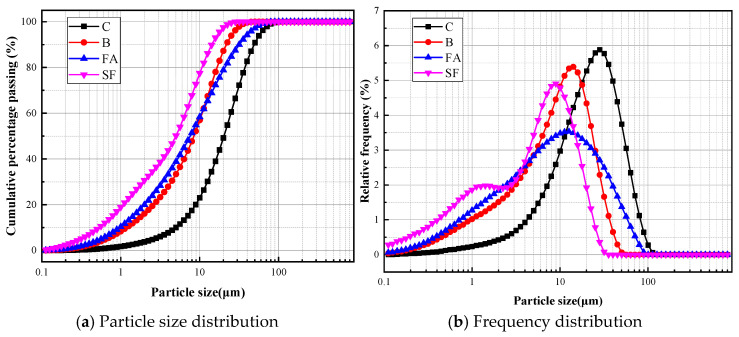
Particle size distribution diagram of raw materials.

The cementitious materials used in the experiment comprised two types: Grade P·O 42.5 ordinary Portland cement (C) produced by Sichuan E’sheng Cement Group Co., Ltd. (E’sheng, Leshan, China), and low-carbon cement (B) manufactured by Shanghai Baioheng New Materials Co., Ltd. (Baioheng, Shanghai, China). This cement belongs to the clinker-free, one-part geopolymer (alkali-activated material). Its raw materials are mainly composed of amorphous or glassy phases derived from industrial solid wastes such as red mud, blast furnace slag, and fly ash. Upon hydration, it primarily forms amorphous aluminosilicate gels (N-A-S-H/C-(N)-A-S-H) along with a small amount of C-S-H gel. The activation mechanism is an alkali-sulfate combined chemical excitation, which requires no high-temperature sintering. After water addition, an alkaline environment (pH > 12) is generated, dissolving Al^3+^ and Si^4+^ from the solid wastes, which then undergo geopolymerization to form a three-dimensional gel network. Aggregates were procured from Sichuan Xinghualutong Renewable Resources Co., Ltd. (Xinghualutong, Chengdu, China), with manufactured sand serving as fine aggregate and recycled aggregate as coarse aggregate. Performance tests conducted on the recycled coarse aggregate yielded the following results: a crushing index of 11.76%, water absorption of 3.9%, apparent density of 2356 kg/m^3^, and bulk density of 1578 kg/m^3^. Its particle size distribution curve is presented in Figure 1. FA (Grade I, supplied by Chengdu Zhongcheng Science and Trade Co., Ltd., Chengdu, China) and SF (commercially available standard product, supplied by Sichuan Langtian Resources Comprehensive Utilization Co., Ltd., Chengdu, China) were used. The chemical compositions of the cement, FA, and SF are listed in Table 1, while their SEM images and particle size distribution diagrams are shown in Figure 2 and Figure 3, respectively.

### 2.2. Experimental Methods

#### 2.2.1. Specimen Preparation

The mix proportions for the low-carbon concrete are outlined in Table 2. A total of six groups of concrete specimens with different mix designs were prepared. The design variables included the types of cement and the replacement levels of supplementary cementitious materials: FA replaced 25% of the cement by mass, and SF replaced 5% of the cement by mass. The water-binder ratio was fixed at 0.3. Detailed mix proportion parameters are listed in Table 2. Specimen preparation followed the designed mix proportions. First, coarse and fine aggregates were dry-mixed for 3 min to ensure uniformity. Cementitious materials were then added and mixed for another 3 min. Water was introduced in three equal portions, with mixing continued for 2 min after each addition, until a homogeneous fresh state was achieved. The mixture was subsequently cast into molds in layers and compacted by vibration. The surfaces of the molds were covered with plastic film to maintain moisture. After 24 h of curing at room temperature, the specimens were demolded and transferred to standard curing conditions until the designated testing ages.

#### 2.2.2. Mechanical Properties Tests

**Figure 4:**
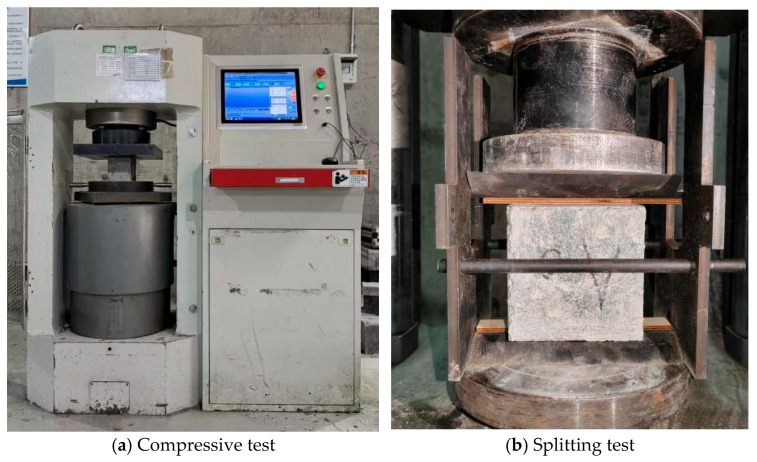
Mechanical property tests.

The mechanical properties of the low-carbon concrete were tested in accordance with the Chinese standard GB/T 50081-2019 [19] “Standard for test methods of mechanical properties of ordinary concrete”, as shown in Figure 4. Both the cube compressive strength and splitting tensile strength tests were conducted using an electro-hydraulic servo universal testing machine with a maximum load capacity of 2000 kN (Shenzhen Wance Testing Machine Co., Ltd., Shenzhen, China). Specimens with dimensions of 100 mm × 100 mm × 100 mm were prepared for testing. The loading rates were set at 0.5 MPa/s for compressive tests and 0.08 MPa/s for splitting tensile tests [20]. All tests were performed in triplicate for each mix proportion, and the results are presented as mean ± standard deviation. The error bars in all figures represent one standard deviation from the mean.

#### 2.2.3. Carbonation Test

**Figure 5:**
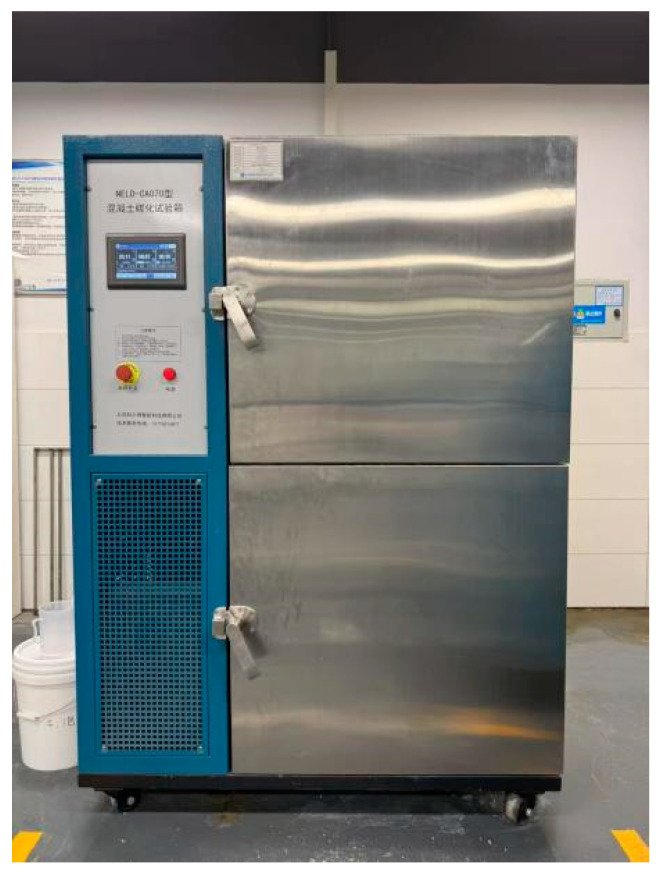
Carbonation test.

The carbonation test was conducted on low-carbon concrete specimens that had been standard-cured for 28 d. The experiment followed the Chinese national standard GB/T 50082-2024 [21] “Standard for test methods of long-term performance and durability of ordinary concrete”. As shown in Figure 5 prior to accelerated carbonation exposure, all 28 d specimens were dried in an oven at 60 °C for 48 h and then cooled to room temperature. Four sides of each specimen were sealed with paraffin wax, leaving two opposite parallel faces exposed to a carbonation chamber. The accelerated carbonation test was conducted under controlled conditions with a CO_2_ concentration of 20 ± 2%, a temperature of 20 ± 2 °C, a relative humidity of 70 ± 5%, and a total exposure duration of 56 d. After 7, 14, 28, and 56 d of CO_2_ exposure, the carbonation depths of three replicate specimens were measured, and the reported results represent the average values. For measurement, each carbonated specimen was split into two halves. The freshly split surface was immediately sprayed with a 1% phenolphthalein ethanol solution. Approximately 30 s later, the carbonation depth was measured at marked intervals of 10 mm using a steel ruler, with at least five measurement points taken per carbonated surface. If a coarse aggregate particle happened to lie exactly on the carbonation front at a measurement point, the average of the carbonation depths on both sides of the particle was taken as the depth for that point. The carbonation depth was measured to an accuracy of 0.5 mm.

#### 2.2.4. Microstructural Test Method

The sample preparation procedures for microstructural characterization were as follows: After the carbonation test and compressive strength test, representative fragments of approximately 10 mm in size were collected from the central region of each specimen and immediately immersed in anhydrous ethanol for 48 h to terminate hydration. The samples were then dried in an oven at 60 °C for 24 h until constant weight was achieved.

Nanoindentation tests were performed using a Hysitron TI980 TriboIndenter (Bruker, Berlin, Germany) equipped with a standard Berkovich tip. The samples for nanoindentation were cut into 10 mm × 10 mm × 10 mm cubes using a low-speed diamond saw (Husqvarna, Stockholm, Sweden), followed by mechanical polishing with diamond pastes down to 0.25 μm and final surface finishing using a 0.05 μm colloidal silica suspension to achieve a mirror-like surface roughness below 5 nm. In this study, a static load-controlled single indentation test method was adopted: the load was linearly increased from 0 mN to 6 mN at a rate of 600 µN/s, held constant at 6 mN for 10 s, and then linearly unloaded from 6 mN back to 0 mN at the same rate. The single-point indentation tests were arranged in an 8 × 8 rectangular grid pattern, with a spacing of 5 μm between adjacent test points along both the X and Y directions, covering a total surface area of 35 μm × 35 μm.

For X-ray diffraction (XRD) analysis, samples were first crushed into small particles using a steel mortar, then ground with an agate mortar to a particle size below 80 μm to ensure sufficient crystallite orientation randomness. The ground powder was then placed into a sample holder and compacted flat. XRD analysis was performed using a Rigaku Ultima IV (Rigaku Corporation, Tokyo, Japan) with Cu Kα radiation (λ = 1.5406 Å) operated at 40 kV and 40 mA, scanning from 5° to 70° (2θ) at a scan rate of 2°/min. For scanning electron microscopy with energy-dispersive X-ray spectroscopy (SEM-EDS) analysis, fragments with particle sizes between 5 mm and 8 mm were selected. These fragments were mounted onto aluminum stubs using carbon conductive tape and sputter-coated with a thin layer of gold (approximately 10 nm) to enhance conductivity and prevent charging effects under electron beam irradiation. SEM-EDS analysis was performed using a ZEISS Sigma 300 (ZEISS, Oberkochen, Germany) equipped with an EDS detector (Oxford Instruments, High Wycombe, UK), operated at an accelerating voltage of 15 kV with a working distance of approximately 10 mm. The EDS detector was used to analyze the elemental composition of selected micro-areas.

## 3. Results and Discussions

### 3.1. Mechanical Property

#### 3.1.1. Compressive Strength

**Figure 6:**
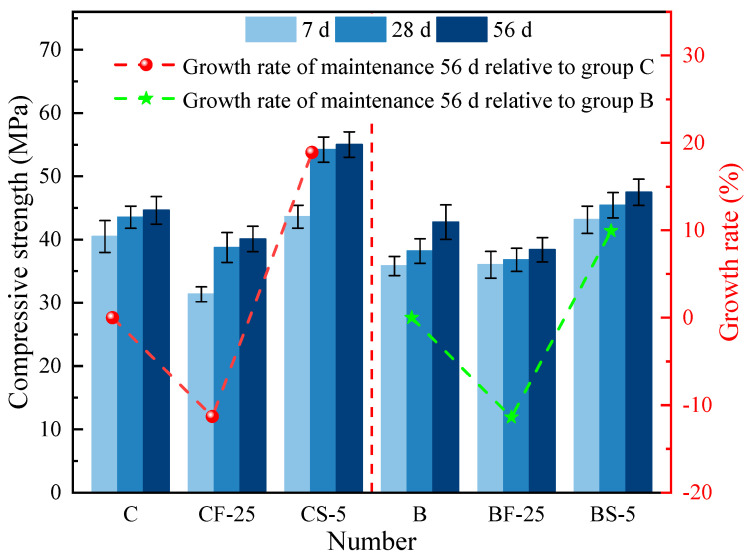
Compressive strength.

Figure 6 presents the compressive strength and its growth rate for different specimens. All tests were performed on three replicate specimens per mix proportion, and the standard deviations of the compressive strength tests ranged from 1.17 MPa to 2.74 MPa, indicating acceptable consistency and reproducibility of the test results. The incorporation of FA into both B-series and C-series low-carbon concrete negatively affected the compressive strength, whereas the addition of SF improved the early-age strength. Specifically, at 56 d of curing, the compressive strength of mix CF-25 decreased by 11.3% compared to that of the C-group, while that of mix CS-5 increased by 18.92% relative to the C-group. Similarly, the strength of mix BF-25 was 11.39% lower than that of the B-group, whereas mix BS-5 showed a 9.94% increase over the B-group. At the same curing age, the compressive strengths of low-carbon concrete containing 25% FA were consistently lower than those of the corresponding control specimens. This result was attributed to the relatively slow pozzolanic reaction of FA, which did not fully participate in the secondary hydration process within the 56 d period, thereby failing to fully utilize its filling effect and pozzolanic activity for strength enhancement. Moreover, the addition of FA reduced the relative content of cement clinker in the system, leading to a decrease in the total amount of early hydration products. At 56 d, the degree of activation of FA remained insufficient to compensate for this strength loss. In contrast, the compressive strengths of low-carbon concrete containing 5% SF consistently exceeded those of the control specimens, which was ascribed to the pozzolanic effect and the micro-aggregate filling effect of SF. A comparison between the B-series and C-series low-carbon concretes revealed that the C-series consistently exhibited higher strengths than the B-series. Furthermore, the rate of strength increase with curing age was more pronounced in the C-series. This difference was due to the fact that the C-series primarily consisted of highly reactive Portland cement clinker, which formed dense and high-strength C-S-H gel during hydration. In contrast, the B-series was based on an activated cementitious system derived from industrial solid wastes; its hydration products possessed a relatively weaker microstructure and lower packing density, ultimately resulting in lower strength compared to the C-series.

#### 3.1.2. Carbonation Test

**Figure 7:**
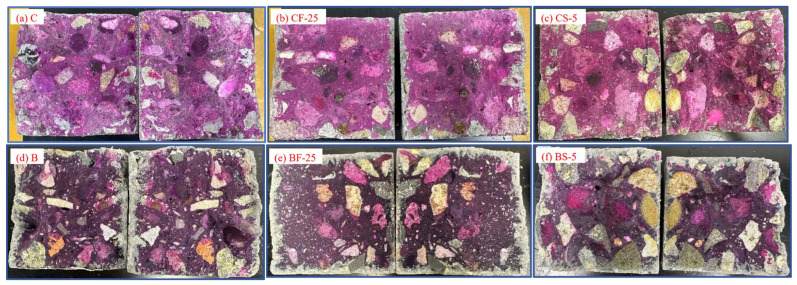
Carbonation for 7 d.

**Figure 8:**
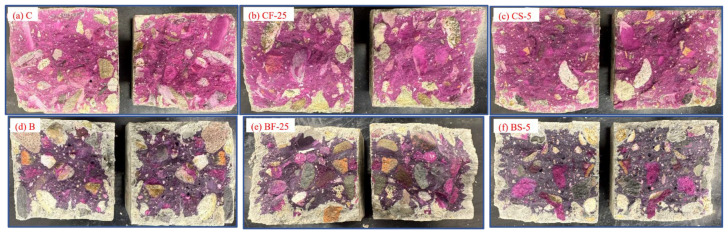
Carbonation for 56 d.

**Figure 9:**
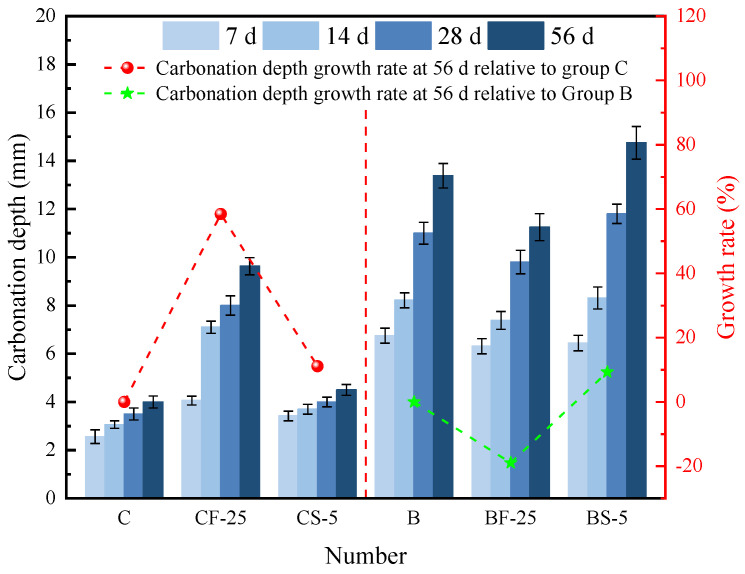
Carbonation depth.

Figure 7 and Figure 8 present the phenolphthalein color-development images of low-carbon concrete after 7 d and 56 d of carbonation, respectively. Figure 9 illustrates the variation in carbonation depth with carbonation age. The carbonation depth error ranges from 0.15 to 0.56 mm, indicating that the measurement error is controlled within a small range and the data are highly reliable. The results showed that the carbonation depth of concrete continued to increase with prolonged exposure. This trend was attributed to the continuous diffusion of carbon dioxide through the pore structure and its subsequent reaction with hydration products. The accumulation of carbonation products gradually drove the carbonation front inward. After 56 d of carbonation, the carbonation depths of the CF-25 and CS-5 mixes increased by 58.46% and 11.11%, respectively, compared to the C-group, while those of the BF-25 and BS-5 mixes changed by −18.93% and 9.29%, respectively, relative to the B-group. A comparison between the B-series and C-series systems revealed that the carbonation depths of the B-series concrete were generally higher than those of the C-series. The primary reason for this difference lies in the hydration products: the C-series concrete generated a substantial amount of calcium hydroxide, establishing a stable, high-alkalinity reserve that could buffer carbon dioxide attack over an extended period. In contrast, the alkalinity of the B-series relied on externally supplied activators; its aluminosilicate gel structure lacked a durable alkaline reserve. Once the surface alkalinity was consumed, the pH dropped rapidly, allowing the carbonation reaction to proceed more easily toward the interior. Moreover, the pore structure of the B-series concrete likely exhibited higher connectivity, providing additional pathways for carbon dioxide diffusion. It is noteworthy that the effect of 25% FA replacement on carbonation depth differed markedly between the C-series and B-series systems. For the C-series concrete, the mix with 25% FA (CF-25) exhibited a significantly higher carbonation depth (an increase of 58.46% relative to the C-group). This phenomenon occurred because the incorporation of FA reduced the clinker content, thereby decreasing the production of calcium hydroxide. Additionally, the slow pozzolanic reaction of FA at early ages was insufficient to fully compensate for the decline in alkalinity reserve through pore refinement and product densification. In contrast, for the B-series concrete, the incorporation of 25% FA (BF-25) led to a reduced carbonation depth (a decrease of 18.93% relative to the B-group). This improvement is likely attributable to the fact that FA particles, under the alkaline activation environment of the B-series system, participated in the geopolymerization reaction, contributing to a denser microstructure and reduced pore connectivity. Consequently, for the C-series, the system remained in a period of relatively weak carbonation resistance during the testing period, whereas the B-series benefited from the incorporation of FA.

**Figure 10:**
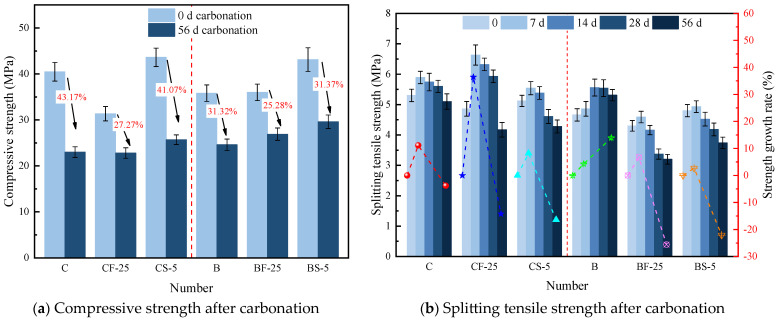
Changes in mechanical properties after carbonation.

Figure 10 illustrates the variations in compressive strength and splitting tensile strength of low-carbon concrete after carbonation. The error range of the compressive strength of carbonated concrete is between 1.05 and 1.48 MPa, and the standard deviation range of the splitting tensile strength is between 0.152 and 0.33 MPa, indicating that the test results exhibit a low degree of dispersion and the data are reliable. After 56 d of carbonation, the compressive strength of all mixes decreased compared to their uncarbonated counterparts: the reductions were 43.17%, 27.27%, 41.07%, 31.32%, 25.28%, and 31.37% for mixes C, CF-25, CS-5, B, BF-25, and BS-5, respectively. In the C-series, the strength decline was primarily attributed to the decalcification and decomposition of C-S-H gel due to its reaction with carbon dioxide, which disrupted the cementitious skeleton and induced micro-cracking from carbonation shrinkage. In the B-series, carbon dioxide attack led to the leaching of alkali ions from the gel network, causing structural de-alkalization, depolymerization, and crystallization/migration of reaction products, ultimately resulting in microstructural degradation. As shown in Figure 10b, the splitting tensile strength initially increased and then decreased with carbonation time. After 7 d of carbonation, the splitting tensile strengths of all mixes increased by 11.13%, 36.42%, 8.20%, 4.29%, 6.74%, and 2.71% relative to their uncarbonated states. However, by 56 d of carbonation, the strengths decreased by 3.77%, 14.20%, 16.41%, 13.95%, 25.58%, and 22.08%, respectively. The early-stage strength increase was attributed to pore-filling by carbonation products such as calcium carbonate, which enhanced surface densification and interfacial bonding. As carbonation progressed, the cumulative damage—stemming from decalcification of C-S-H gel in the C-series and depolymerization of the gel network in the B-series, coupled with the development of carbonation-shrinkage micro-cracks—gradually outweighed the initial pore-filling effect, leading to the eventual decline in splitting tensile strength. The macroscopic experimental data are summarized in Table 3.

### 3.2. Microstructural Analysis

#### 3.2.1. Nanoindentation Test

**Figure 11:**
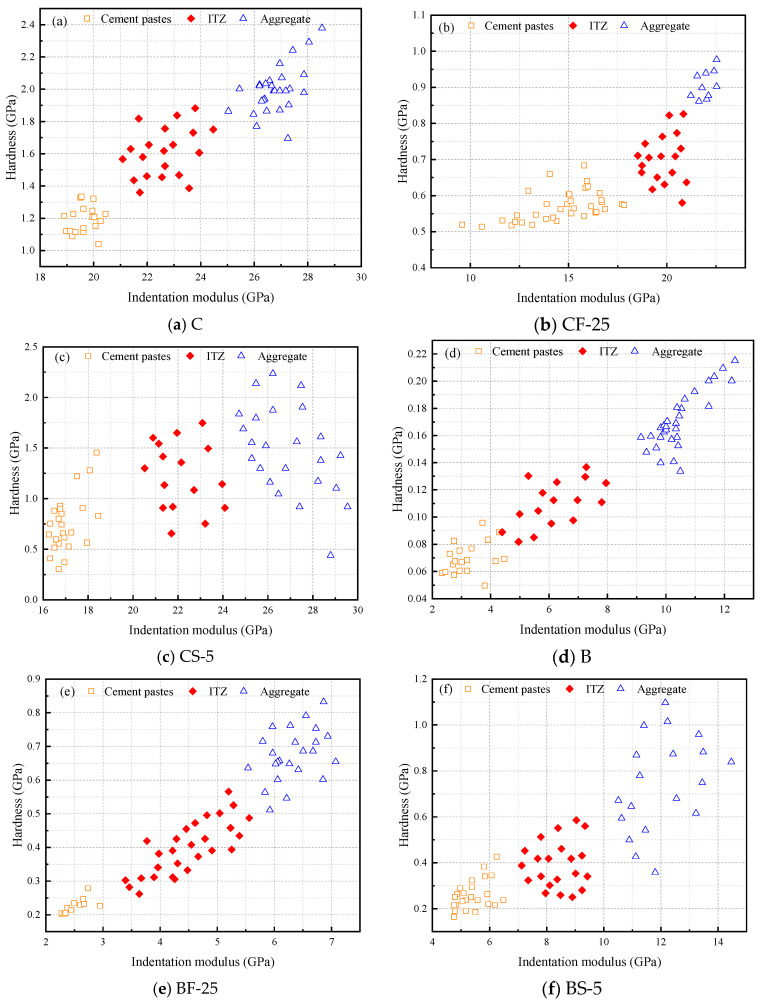
Distribution of microscopic mechanical properties of different samples.

**Figure 12:**
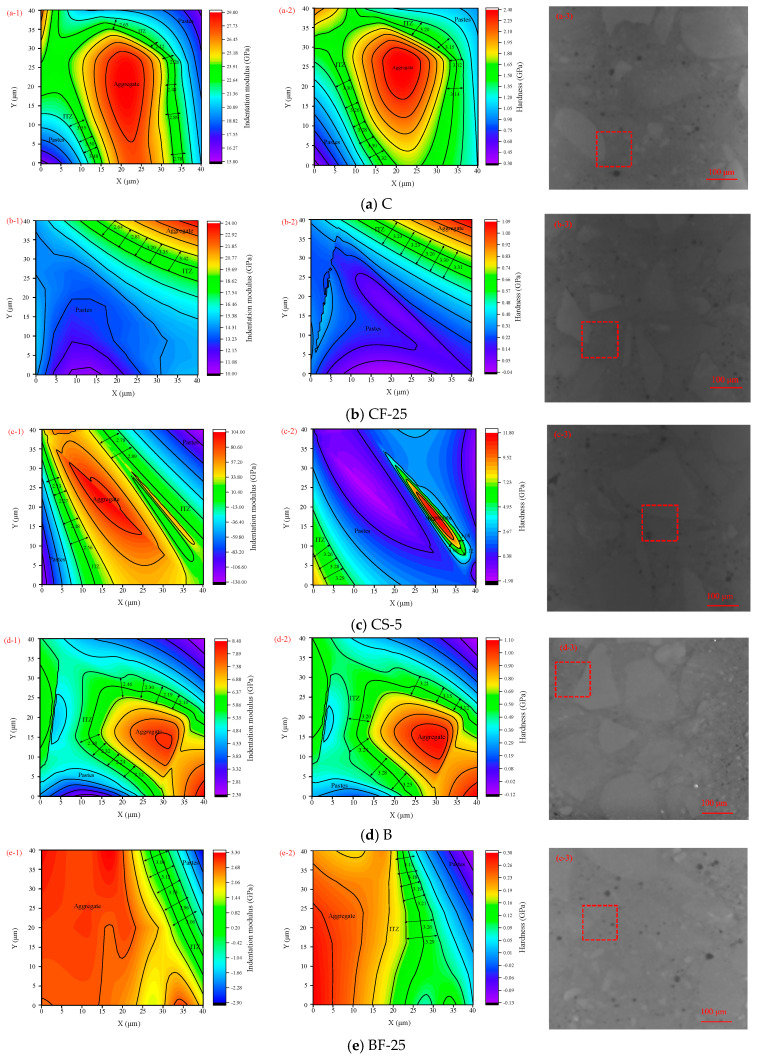

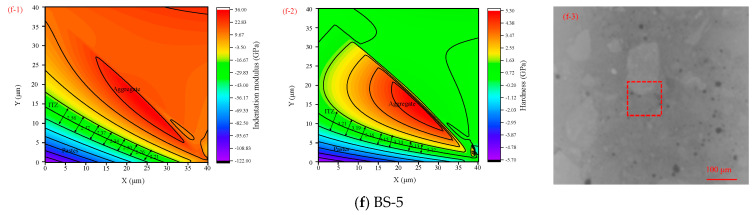
Contour maps of indentation results for low-carbon concrete.

Figure 11 presents the distribution of indentation modulus for different specimens. For the C-group (Figure 11a), the modulus ranges of the cement paste, ITZ, and aggregate were 18.90–20.45 GPa, 21.08–24.47 GPa, and 25.03–28.53 GPa, respectively. For the CF-25 group (Figure 11b), the corresponding ranges were 10.57–17.83 GPa, 18.52–21.01 GPa, and 21.55–22.53 GPa. For the CS-5 group (Figure 11c), the ranges were 16.25–18.45 GPa, 20.52–24.09 GPa, and 24.72–29.55 GPa. Meanwhile, for the B-group (Figure 11d), the three phases exhibited modulus ranges of 2.33–4.47 GPa, 4.39–7.95 GPa, and 9.15–12.36 GPa. For the BF-25 group (Figure 11e), the ranges were 2.27–2.95 GPa, 3.39–5.56 GPa, and 5.54–7.07 GPa; and for the BS-5 group shown in Figure 11f, the ranges were 4.75–6.49 GPa, 7.12–9.43 GPa, and 10.51–14.47 GPa, respectively. To investigate the thickness of the ITZ, contour maps of indentation modulus and hardness distribution were plotted for the C, CF-25, CS-5, B, BF-25, and BS-5 specimens, as shown in Figure 12. The indentation modulus and hardness of the ITZ lay between those of the cement paste and the aggregate, with the green regions in the figure representing the ITZ. The color gradient from blue to red indicates the transition from low to high elastic modulus, revealing the differences in mechanical properties among the distinct phases. In Figure 12, (x-1) represented the indentation modulus; (x-2) represented the hardness; (x-3) represented the schematic diagram of the nanoindentation test area. The variable x corresponded to designations a through f.

**Figure 13:**
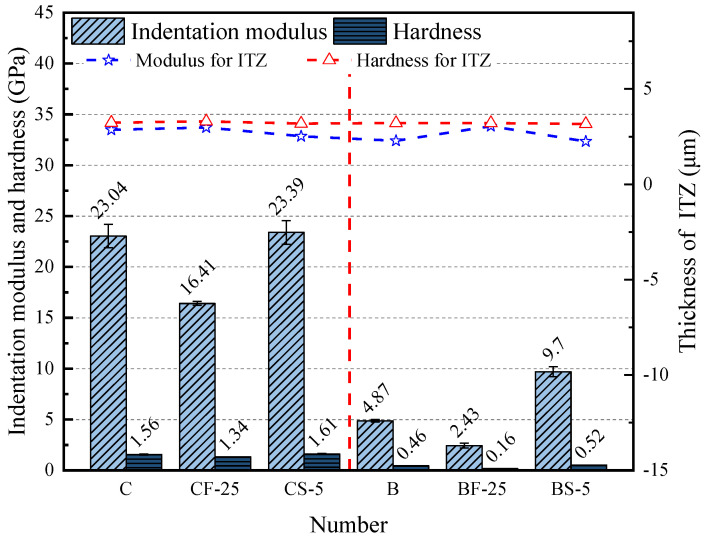
The key parameters of the low-carbon green concrete were obtained through the nanoindentation method.

As shown in Figure 13, the average indentation modulus values for C, CF-25, CS-5, B, BF-25, and BS-5 were 23.04, 16.41, 23.39, 4.87, 2.43, and 9.7 GPa, respectively, while the average indentation hardness values were 1.56, 1.34, 1.61, 0.46, 0.16, and 0.52 GPa, respectively. Furthermore, the replacement of cement with FA increased the thickness of the ITZ, whereas the replacement with SF reduced it. Specifically, compared to C, the thickness of CF-25 increased by 0.07–0.12 μm, while that of CS-5 decreased by 0.025–0.34 μm; compared to B, the thickness of BF-25 increased by 0–0.78 μm. The average ITZ thickness in CS-5 and BS-5 decreased by 0.025–0.34 μm and 0.048–0.05 μm, respectively. After replacing 25% of ordinary Portland cement with FA, the nanoindentation hardness and modulus of the material decreased, and the ITZ thickness increased. This outcome was attributed to the low early-age pozzolanic activity of FA, which slowed the hydration process, leading to insufficient formation of early hydration products such as C-S-H gel. Consequently, the ITZ exhibited a looser microstructure, higher porosity, and accumulation of unreacted FA particles that created localized weak zones. These factors collectively impaired the early-age mechanical performance and enlarged the interfacial transition zone. In contrast, the incorporation of SF significantly improved the mechanical properties of the ITZ and reduced its thickness, demonstrating superior effectiveness in enhancing micromechanical performance and refining the ITZ compared to FA, which is consistent with previous research findings [22].

The pozzolanic reaction of SF consumed Ca(OH)_2_ to form additional C-S-H gel, increasing the packing density of the ITZ. Meanwhile, the fine SF particles physically filled the capillary pores and microvoids, thereby reducing the thickness and mechanical gradient across the ITZ. The mechanism by which the addition of SF enhances the ITZ in concrete is as follows: on the one hand, the pozzolanic reaction converted the calcium hydroxide (CH) near the aggregates into more stable and higher-strength calcium silicate hydrate (C-S-H) gel; on the other hand, the micro-fine particles filled the excessive pores within the ITZ [23,24].

#### 3.2.2. XRD Analysis

**Figure 14:**
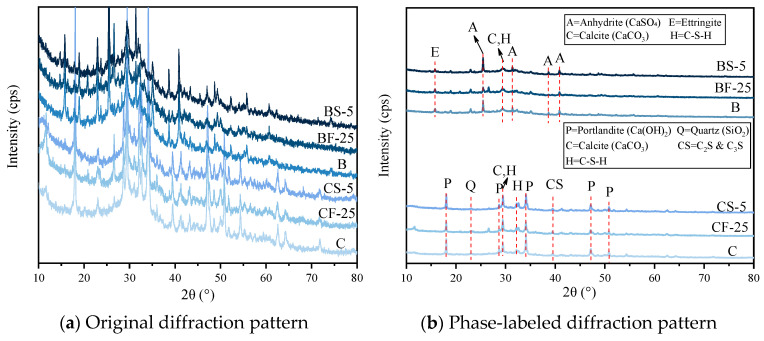
XRD analysis.

Figure 14 presents the XRD phase analysis results for Group C and Group B, along with their respective admixture systems. As shown in the figure, the hydration products of Group C exhibited well-crystallized Ca(OH)_2_, C-S-H, and CaCO_3_, along with minor amounts of C_2_S and C_3_S. Characteristic peaks for Ca(OH)_2_ were identified at 2θ values of approximately 18.09°, 28.75°, 34.10°, 47.25°, and 50.86°. Characteristic peaks for C-S-H and CaCO_3_ were observed near 29.37° and 32.22°, respectively. Characteristic peaks for C_2_S and C_3_S were visible at 39.54°. In contrast, distinct Ca(OH)_2_ characteristic peaks were not observed in Group B, indicating that its hydration process differed significantly from that of ordinary cement. This system exhibited a diffraction peak for ettringite near 15.79°, while a composite diffraction peak for C-S-H and CaCO_3_ was observed at 29.37°. This suggested that the pozzolanic reaction dominated in this system, consuming the generated calcium hydroxide during hydration, which promoted the formation of additional C-S-H gel and ettringite. Simultaneously, a certain degree of carbonation occurred in the system. Regarding the influence of admixtures, with an FA content of 25%, the diffraction peak intensities in Groups C and B decreased. This was attributed to the relatively low early-age pozzolanic activity of FA. During the initial hydration period, FA primarily functioned as a physical filler and diluent, reducing the total amount and crystallinity of the early hydration products. Consequently, this led to a decrease in macroscopic strength and a weakening of the diffraction signals. With an SF content of 5%, the diffraction peak intensities in Groups C and B increased. This was due to the extremely high specific surface area and pozzolanic activity of SF. The nano-sized particles not only effectively filled microscopic pores but also rapidly reacted with free Ca(OH)_2_ in the system. This significantly promoted the generation and densification of hydration products such as C-S-H gel, thereby enhancing the macroscopic strength and intensifying the diffraction peaks of the corresponding products. The increased SF content elevated the Si/Al ratio of the hydration products, as reflected in the XRD peak shifts and intensity variations, indicating a higher degree of polymerization of the C-(N)-A-S-H gel network. The absence of Ca(OH)_2_ peaks in the B-series confirmed that the low-calcium system relies on alkali-activated geopolymerization rather than Portland-like hydration, which fundamentally changes the carbonation mechanism and ITZ bonding behavior.

#### 3.2.3. Sem-Eds Analysis

**Figure 15:**
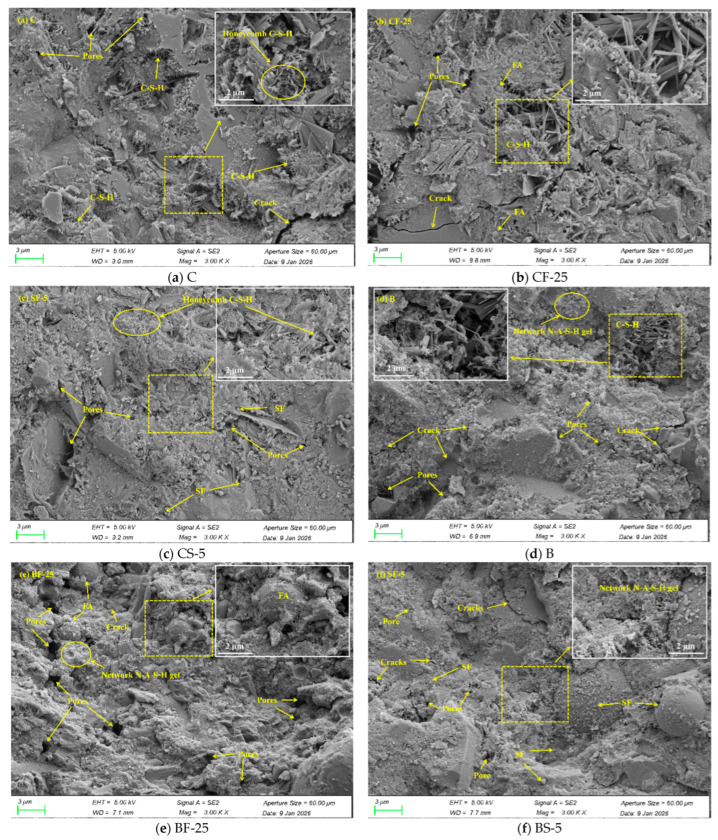
SEM analysis of different concrete samples.

In low-carbon concrete, the microstructure of the ITZ, particularly the presence of pores and microcracks, significantly influenced the stiffness and elastic modulus of the concrete. The ITZ morphology was analyzed via SEM. As shown in Figure 15, the control group C exhibited a relatively dense structure rich in C-S-H gel, with few pores and cracks. Group CF-25 displayed a loose structure with unreacted FA particles and numerous pores. Group CS-5 showed a more compact structure. Within Group B, the reference sample revealed needle-shaped C-S-H and a network of N-A-S-H gel. Group BF-25 exhibited a significant increase in porosity. In contrast, Group BS-5, due to the filling effect of SF, produced a large amount of N-A-S-H gel, resulting in a relatively dense structure. The results indicated that at an FA replacement rate of 25%, incomplete hydration reactions led to a decrease in compressive strength. However, the FA particles filled microcracks and pores, optimizing the pore structure. At an SF replacement rate of 5%, mechanical properties improved, and the microstructure became denser. However, due to its high specific surface area and high reactivity, local areas were prone to form insufficiently reacted SF agglomerates and interconnected pores. Comparing the C and B systems, the N-A-S-H gel in Group B exhibited weaker chemical bonding with the aggregates. Furthermore, it lacked the interfacial filling and strengthening effects provided by crystalline phases such as CH, resulting in insufficient bonding within the ITZ region, higher porosity, and a lower degree of hydration.

**Figure 16:**
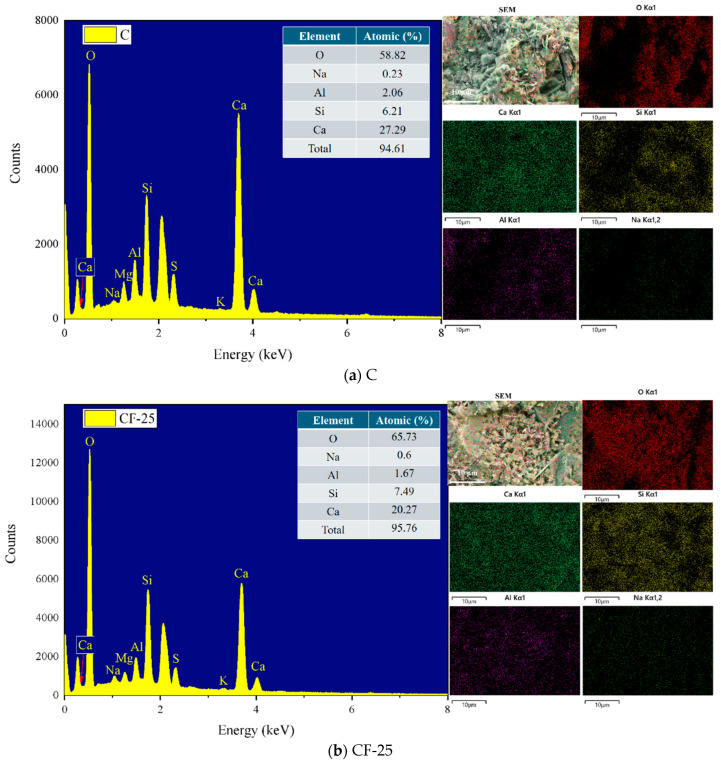

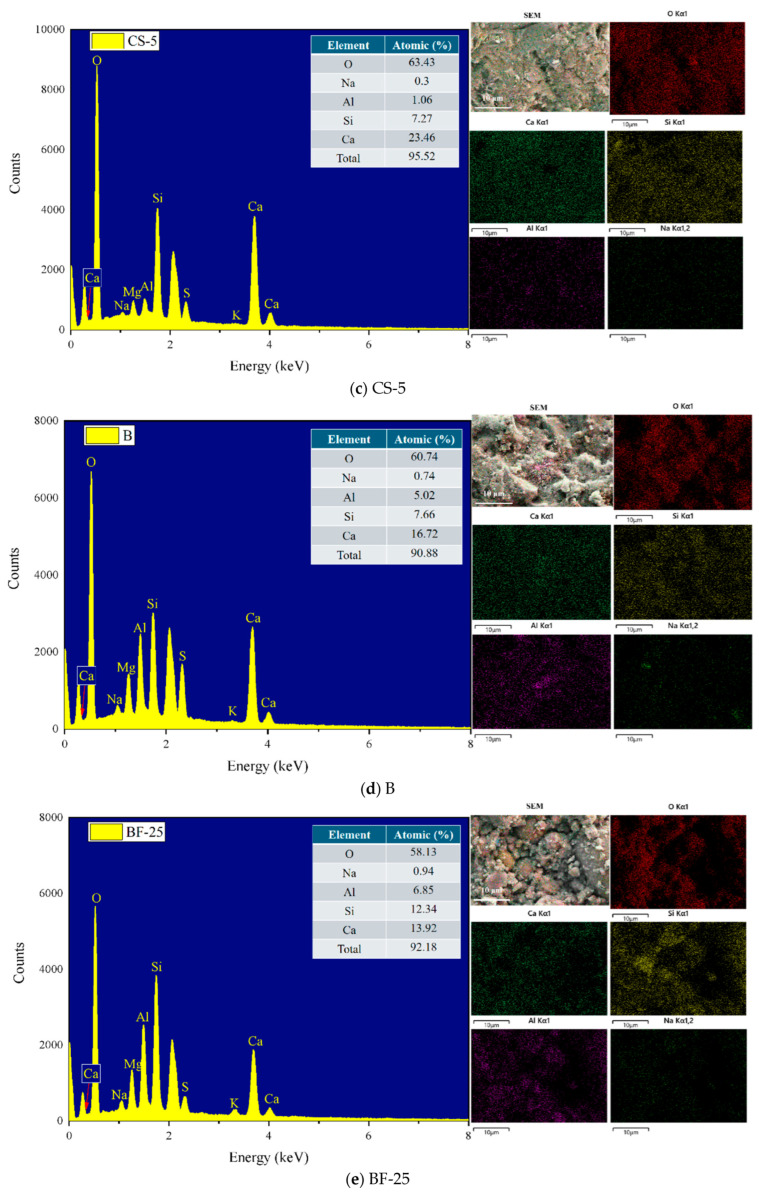

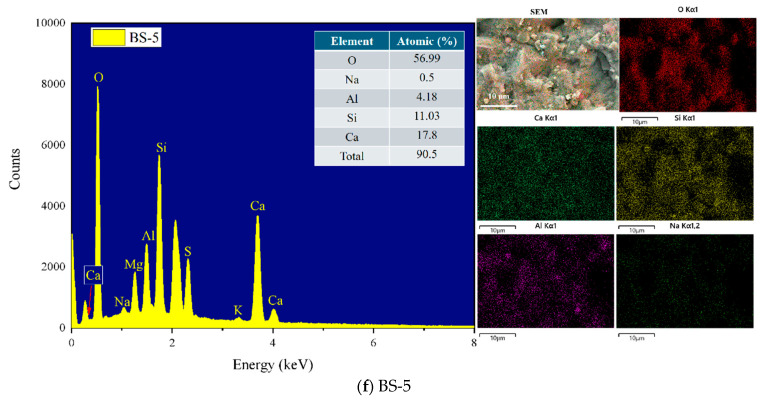
SEM-EDS analysis of different specimens.

The quantitative elemental composition and distribution results for the paste of low-carbon concrete are presented in Figure 16 and Table 4, respectively. The elemental ratios of the reaction products ranged as follows: Si/Al = 1.53–6.86, Na/Al = 0.04–0.10, Ca/Si = 1.13–4.39, and Na/Si = 0.04–0.10. This indicated a potential coexistence of N-A-S-H and C–S–H phases within the microstructure, which was consistent with the XRD analysis results. Specifically, Group C exhibited a Ca/Si ratio of 4.39. With a 25% FA incorporation (CF-25), this ratio decreased to 2.71, and with a 5% SF incorporation (CS-5), it was 3.23. In Group B, the Ca/Si ratio was 2.18, which decreased to 1.13 with 25% FA (BF-25) and was 1.61 with 5% SF (BS-5). The Si/Al ratios for the groups were as follows: 3.01 for Group C, 4.49 for Group CF-25, 6.86 for Group CS-5, 1.53 for Group B, 1.80 for Group BF-25, and 2.64 for Group BS-5. The aforementioned data demonstrated that the incorporation of both FA and SF significantly altered the chemical composition of the hydration products. In the C system, FA decreased the Ca/Si ratio and increased the Si/Al ratio, reflecting the dilution and consumption of calcium by its silicoaluminous components. SF, on the other hand, substantially increased the Si/Al ratio while decreasing the Ca/Si ratio, indicating the strong regulating effect of its highly reactive SiO_2_ on the system. In the B system, due to its inherently low base Ca/Si and Si/Al ratios, the incorporation of SCMs further decreased the Ca/Si ratio, with a slight increase in Si/Al, although these values remained significantly lower than those in the C system. This was consistent with the reaction characteristics dominated by low-calcium geopolymeric gel. The physical mechanism of FA is primarily dilution and microfilling at early ages, with its chemical pozzolanic effect becoming significant only after prolonged curing. This delayed reactivity explains the lower Ca/Si ratio and higher porosity in FA-blended samples. In contrast, SF provides immediate physical filling and rapid chemical consumption of portlandite, leading to higher Si/Al ratios, greater gel network polymerization, pore refinement, and enhanced ITZ mechanical properties. The absence of Ca(OH)_2_ peaks in the B-series confirms that the low-calcium system relies on alkali-activated geopolymerization, which fundamentally alters the carbonation mechanism and ITZ bonding behavior. Changes in chemical composition were closely related to macroscopic compressive strength. Due to its low early-age reactivity, FA led to a slow formation of hydration products and insufficient densification of the microstructure, resulting in a decline in strength. In contrast, SF, through its high reactivity and micro-filling effect, promoted the formation of a dense, highly polymerized gel structure while modulating the chemical composition, thereby enhancing strength. This suggested that the influence of SCMs on strength depended not only on changes in chemical composition but also critically on their reaction kinetics and the efficiency of microstructure formation.

## 4. Conclusions

Based on the systematic durability experiments and multi-scale analysis of industrial solid waste-based low-carbon concrete conducted in this study, the following conclusions were drawn:

(1) The use of low-carbon concrete is justified when supplementary cementitious materials are properly selected. The addition of 25% FA significantly reduces compressive strength, whereas the incorporation of 5% SF effectively enhances strength, demonstrating that SF is a more suitable modifier for maintaining mechanical performance in low-carbon concrete.
(2) Regarding durability, FA substantially increases carbonation depth due to reduced alkali reserve and slow early-age reactivity, while SF has a limited adverse effect. The low-calcium alkali-activated system (B-series) exhibits inherently higher carbonation susceptibility compared to the high-calcium Portland system (C-series), indicating that additional carbonation protection may be needed for low-calcium low-carbon concrete in service.
(3) From a microstructural perspective, SF significantly improves the mechanical properties of the interfacial transition zone and reduces its thickness through high pozzolanic activity and nano-filling effects, whereas FA shows limited effectiveness due to its delayed reactivity.
(4) Overall, the performance of low-carbon concrete is governed not only by the chemical composition changes induced by the admixtures but also critically by their reaction kinetics and microstructure formation efficiency. SF at 5% is an effective strategy to balance mechanical strength, carbonation resistance, and microstructural densification, while FA at 25% requires careful consideration or additional mitigation measures in carbonation-prone environments.

## 5. Future Perspectives

While this study provides fundamental insights into the carbonation behavior of low-carbon concrete incorporating FA and SF, certain limitations should be addressed in future research. The accelerated carbonation exposure in this work was limited to 56 days, which, although consistent with standard protocols, may not fully capture long-term carbonation progression under natural service conditions where environmental parameters vary dynamically. Therefore, extended natural carbonation tests spanning longer durations are recommended to establish more reliable time-dependent carbonation models and to calibrate accelerated-to-natural carbonation conversion factors. Additionally, this study employed only one replacement level for each additive (25% FA and 5% SF). The performance of FA and SF is likely dosage-dependent, and optimal mix designs may exist at different replacement ratios. Future investigations should systematically examine a broader range of FA and SF dosages to identify the optimal balance between mechanical properties, carbonation resistance, workability, and economic feasibility. Addressing these perspectives will further advance the large-scale engineering application and standardization of low-carbon concrete.

## Figures and Tables

**Table 1:** Chemical composition of cement, fly ash, and silica fume (wt%).

Samples	SiO_2_	K_2_O	MgO	Na_2_O	Fe_2_O_3_	SO_3_	Al_2_O_3_	CaO	Others
C	24.82	0.51	3.27	1.46	2.49	5.07	8.85	51.75	1.78
B	28.42	0.452	7.95	0.838	0.703	12.56	18.06	29.93	1.087
FA	50.25	1.67	3.53	4.57	4.12	1.2	26.05	7.19	1.42
SF	86.71	3.3	3.19	2.36	1.3	0.821	0.778	0.753	0.788

**Table 2:** Mix proportions of the tested concretes.

Number	C (kg/m^3^)	B (kg/m^3^)	FA (kg/m^3^)	SF (kg/m^3^)	Sand (kg/m^3^)	Recycled Coarse Aggregate (kg/m^3^)	Water (kg/m^3^)	Water-Reducing Admixture (kg/m^3^)
C	500	0	0	0	678	1106	150	2
CF-25	375	0	125	0	678	1106	150	2
CS-5	475	0	0	25	678	1106	150	2
B	0	500	0	0	678	1106	150	2
BF-25	0	375	125	0	678	1106	150	2
BS-5	0	475	0	25	678	1106	150	2

Note: C denotes ordinary portland cement, B denotes low-carbon cement; CF indicates the addition of FA, while CS indicates the addition of SF. For example, CF-25 represents 25% cement replacement by FA, and BF-25 represents 25% low-carbon cement replacement by FA.

**Table 3:** Macroscopic experimental results summary.

Test Name		Number	C	CF-25	CS-5	B	BF-25	BS-5
	Age (d)							
Standard cured compressive strength (MPa)	7		40.47	31.35	43.61	35.82	36	43.13
	28		43.53	38.74	54.21	38.19	36.78	45.41
	56		44.61	40.08	55.02	42.75	38.38	47.47
Compressive strength after carbonation (MPa)	56		23	22.8	25.7	24.6	26.9	29.6
Splitting tensile strength after carbonation (MPa)	0		5.3	4.86	5.12	4.66	4.3	4.8
	7		5.89	6.63	5.54	4.86	4.59	4.93
	14		5.74	6.32	5.38	5.56	4.16	4.52
	28		5.6	5.93	4.61	5.54	3.37	4.18
	56		5.1	4.17	4.28	5.31	3.2	3.74
Carbonation depth (mm)	7		2.56	4.06	3.42	6.75	6.31	6.44
	14		3.06	7.1	3.7	8.22	7.38	8.31
	28		3.5	8	4	11	9.8	11.8
	56		4	9.63	4.5	13.38	11.25	14.75

**Table 4:** Average elemental component results of representative EDS spectra from Figure 16.

Number	Element Ratio			
	Ca/Si	Si/Al	Na/Si	Na/Al
C	4.39	3.01	0.04	0.11
CF-25	2.71	4.49	0.08	0.36
CS-5	3.23	6.86	0.04	0.28
B	2.18	1.53	0.10	0.15
BF-25	1.13	1.80	0.08	0.14
BS-5	1.61	2.64	0.05	0.12

## Data Availability

The original contributions presented in this study are included in the article. Further inquiries can be directed to the corresponding author.

## References

[1] Jiang Z., Zhu Z., Fang D., Fu C., Li S., Jing Y. (2026). Multi-Dimensional Assessment of Low-Carbon Engineering Cement-Based Composites Based on Rheological, Mechanical and Sustainability Factors. Materials.

[2] Wang F., Hua J., Xue X., Wang N., Yan F., Feng D. (2023). Effect of Superfine Cement Modification on Properties of Coral Aggregate Concrete. Materials.

[3] Zhan J., Li H., Li H., Cheng Z., Fu B. (2022). Composition Design and Characterization of Alkali-Activated Slag–Metakaolin Materials. Front. Built Environ..

[4] Yue W. (2021). Reducing carbon emissions, solid waste recycling has great potential. China Building Materials News.

[5] Zhan J., Fu B., Cheng Z. (2022). Macroscopic Properties and Pore Structure Fractal Characteristics of Alkali-Activated Metakaolin—Slag Composite Cementitious Materials. Polymers.

[6] Nazeer M., Kapoor K., Singh S.P. (2023). Strength, Durability and Microstructural Investigations on Pervious Concrete Made with Fly Ash and Silica Fume as Supplementary Cementitious Materials. J. Build. Eng..

[7] Chen B., Rao M., Feng Y. (2024). Effects of Curing Temperature and Supplementary Cementitious Materials on the Interfacial Transition Zone (ITZ) of High-Ferrite Cement Products. Constr. Build. Mater..

[8] Shelote K.M., Bala A., Gupta S. (2023). An Overview of Mechanical, Permeability, and Thermal Properties of Silica Fume Concrete Using Bibliographic Survey and Building Information Modelling. Constr. Build. Mater..

[9] Wang X., Li X., Zhong Y., Li H., Wang J. (2024). Properties and Microstructure of an Interfacial Transition Zone Enhanced by Silica Fume in Concrete Prepared with Coal Gangue as an Aggregate. ACS Omega.

[10] Siddique R. (2011). Utilization of Silica Fume in Concrete: Review of Hardened Properties. Resour. Conserv. Recycl..

[11] Lv X., Shi Y., Zhou S. (2025). Effects of Fly Ash and Silica Fume on Abrasion Resistance of High-Ferrite Portland Cement: A Comparative Study. Constr. Build. Mater..

[12] Wang F., Sun X., Tao Z., Pan Z. (2022). Effect of Silica Fume on Compressive Strength of Ultra-High-Performance Concrete Made of Calcium Aluminate Cement/Fly Ash Based Geopolymer. J. Build. Eng..

[13] Chen X.-F., Lu C., Xian X., Bian L., Ng S.T. (2026). Degradation Behaviour of Marine Concrete with Mineral Admixtures under the Coupled Effect of Multi-Ionic Attack and Freeze-Thaw Deterioration. Case Stud. Constr. Mater..

[14] Ghazouani N., Salmi A., Alawi Al-Naghi A.A., Elhadi K.M., Raza A. (2026). Engineering Properties of Municipal Solid Waste Incineration Fly Ash-Based Thermally Cured Ultra-High-Performance Green Cement Composites. Ceram. Int..

[15] Qiu J., Zhang T., Li L., Huo Y., Lei T., Liu Y. (2025). Improvement of Mechanical Properties and Frost Resistance of Coal Gangue Concrete: Synergistic Effect and Microscopic Mechanism of Basalt Fiber, Fly Ash and Silica Fume. J. Clean. Prod..

[16] Sabtiwu M., Dhandapani Y., Drewniok M., Adu-Amankwah S., Bernal S.A. (2025). Carbonation Induced Changes in the Mechanical Performance, Water and Chloride Permeability of Portland Cement-Slag-Limestone Ternary Cement Concretes. Cem. Concr. Compos..

[17] Liu J., Liu Y., Zeng J., Zhuge Y. (2025). A Comprehensive Review of Mechanisms, Techniques, and Precursors in Enforced Carbonation for Low-Carbon Concrete. J. Build. Eng..

[18] (2017). Recycled Coarse Aggregate for Concrete.

[19] (2019). Standard for Test Methods of Concrete Physical and Mechanical Properties.

[20] Xue X., Wang F., Hua J., Wang N., Huang L., Chen Z., Yao Y. (2022). Effects of Polyoxymethylene Fiber on Fresh and Hardened Properties of Seawater Sea-Sand Concrete. Polymers.

[21] Ministry of Housing and Urban-Rural Development (2024). Standard for Test Methods of Long-Term Performance and Durability of Ordinary Concrete.

[22] Nežerka V., Bílý P., Hrbek V., Fládr J. (2019). Impact of Silica Fume, Fly Ash, and Metakaolin on the Thickness and Strength of the ITZ in Concrete. Cem. Concr. Compos..

[23] Megat Johari M.A., Brooks J.J., Kabir S., Rivard P. (2011). Influence of Supplementary Cementitious Materials on Engineering Properties of High Strength Concrete. Constr. Build. Mater..

[24] Gholampour A., Ozbakkaloglu T. (2017). Performance of Sustainable Concretes Containing Very High Volume Class-F Fly Ash and Ground Granulated Blast Furnace Slag. J. Clean. Prod..

